# Burden of *Mycoplasma genitalium* and bacterial co-infections in a population-based sample in New Mexico

**DOI:** 10.1097/OLQ.0000000000001472

**Published:** 2021-12-01

**Authors:** Anne Hammer, Patti E. Gravitt, Rachael Adcock, Nicole Patterson, Jack Cuzick, Cosette M. Wheeler, on behalf of the New Mexico HPV Pap Registry Steering Committee

**Affiliations:** 1 Department of Obstetrics and Gynecology, Gødstrup Hospital, Gl. Landevej 61, 7400 Herning, Denmark,; 2 Department of Clinical Medicine, Aarhus University, Palle Juul Jensens Boulevard 99, 8200 Aarhus N, Denmark,; 3 Department of Obstetrics and Gynecology, Aarhus University Hospital, Denmark; 4 Department of Epidemiology and Public Health, University of Maryland School of Medicine, 660 W. Redwood St, Howard Hall 102-B, Baltimore, MD 21201, USA,; 5 Wolfson Institute of Preventive Medicine, Queen Mary University of London, Charterhouse Square, London EC1M 6BQ, UK,; 6 Department of Pathology, University of New Mexico Health Sciences Center, MSC02-1670 House of Prevention Epidemiology, Albuquerque, NM 87131, USA,; 7 Department of Obstetrics and Gynecology, University of New Mexico Health Sciences Center, MSC02-1670 House of Prevention Epidemiology, Albuquerque, NM 87131, USA

**Keywords:** *Mycoplasma genitalium*, sexually transmitted infections, prevalence, epidemiology, cervical cytology

## Abstract

In this population-based US study, the overall prevalence of *Mycoplasma genitalium* was 1.95% (95% CI 1.62–2.34), declining from 6.12% (95% CI 4.72 to 7.92) in women aged 21–24 to 0.48% (95% CI 0.25–0.94) in women aged 40–64. The prevalence of co-infections with *Chlamydia trachomatis* and *Trichomonas vaginalis* was low.

## Short summary

The prevalence of *Mycoplasma genitalium* (Mgen) among women aged 21–24 was similar to that of *Chlamydia trachomatis* (CT), for which women are screened regularly in the US.

## Introduction

Sexually transmitted infections (STI) are a significant burden of disease in the US, with an estimated 19.7 million incident infections in 2008 (1). While most STI’s are asymptomatic or cause transient inflammation, some STI’s, such as *Chlamydia trachomatis* and *Neisseria gonorrhea*, may be associated with significant reproductive harms (2, 3).

Accumulating evidence suggests that *Mycoplasma genitalium*, a common cause of male urethritis (4), may be associated with cervicitis, endometritis, pelvic inflammatory disease, and possibly tubal factor infertility in women (4–8). While screening for *Chlamydia trachomatis* may effectively prevent pelvic inflammatory disease (PID) and subsequent infertility (9), the benefit of screening high-risk women for *Mycoplasma genitalium* remains unclear and controversial (8). This may be because of limited knowledge on the potential reproductive harms associated with an asymptomatic infection, and because the burden of *Mycoplasma genitalium* infections at the population level in the US remains unclear. We therefore aimed to describe the overall and age-specific prevalence of *Mycoplasma genitalium,* and the proportion of cases co-infected with other bacterial STI’s in a population-based sample.

## Methods

We conducted a population-based, cross-sectional study using liquid-based cytology (LBC) residuals, with selections performed through collaboration with the New Mexico HPV Pap Registry (NMHPVPR).

Samples were obtained from three volunteering laboratories who perform cervical screening tests on approximately 70% of the women receiving cervical cancer screening state-wide (80% screening coverage (10)). Following routine clinical testing, discarded LBC residuals were de-identified and subsequently stored at 4°C until testing. Using the NMHPVPR data resource, an age- and cervical cytology stratified random sample of residual LBC specimens was selected for STI analysis (Table 1). This study was deemed exempt from approval by the UNM Human Research Review Committee.

The study population consisted of women who attended cervical cancer screening in New Mexico from August 1^st^ 2013 through July 31^st^ 2014. The sampling design was set up with the intention of oversampling younger women and women with abnormal cervical cytology to allow future estimation of cervical intraepithelial neoplasia risk associated with prevalent STI’s (Table 1). Women were eligible for final analysis if they were aged 21–64 and had valid test results for *Chlamydia trachomatis, Mycoplasma genitalium, Trichomonas vaginalis*, *Neisseria gonorrhea,* and *human papillomavirus (HPV).*

From each LBC sample, we removed 1 mL and placed in an Aptima specimen transport kit (Hologic, cat. no. 301154c) according to the *in vitro* diagnostics product insert. Samples subsequently underwent testing at our University of New Mexico research laboratory for *Chlamydia trachomatis* and *Neisseria gonorrhea* using the FDA (US Food and Drug Administration)-approved Aptima Combo2^®^ assay (Hologic), for *Trichomonas vaginalis* using the FDA-approved Aptima^®^ Trichomonas vaginalis assay (Hologic), and for HPV using the FDA-approved Aptima^®^ HPV assay (Hologic). Testing for *Mycoplasma genitalium* was performed using a prototype of the Aptima^®^ Mycoplasma genitalium assay (Hologic), which received FDA approval January 23, 2019. All STI analyses were performed on the Panther^®^ platform, a fully automated system, following the manufacturer’s instructions. Results were recorded as positive, negative, invalid, or equivocal (*Chlamydia trachomatis* only). Invalid test results were excluded from final analysis as missing (n=297). Equivocal results on *Chlamydia trachomatis* (n=1) were considered positive.

We calculated weighted population prevalence estimates of *Mycoplasma genitalium*, including 95% confidence intervals (CI), overall and by age (21–24, 25–30, 31–34, 35–39, and 40–64 years) (Table 1). Estimates were calculated by weighting back to 84,686 women with an available LBC residual. Weighted logistic models were fit to test for trend in prevalence with increasing age. The sampling fractions and weights (inverse of the sampling fractions) were adjusted to reflect missing data. Additionally, we calculated the prevalence of pair-wise co-infections between *Mycoplasma genitalium* and *Chlamydia trachomatis* and between *Mycoplasma genitalium* and *Trichomonas vaginalis*. Analyses were conducted in Stata version 13 (StataCorp. College Station, TX) using the Survey (SVY) commands.

## Results

A total of 84,686 residual LBC samples from New Mexico laboratories were available for testing, from which 7,867 (9.3%) were originally selected for STI analysis using stratified random sampling (Table 1). From these, we excluded samples from women outside of the age range for routine cervical screening <21 (n=297) and >64 years (n=305) and samples with incomplete STI panel results (missing sample aliquots (n=111), invalid test runs with one or more STI panel (n=217)) leaving 7,057 with complete test results in the final analysis.

The overall weighted population prevalence of *Mycoplasma genitalium* was 1.95% (95% CI 1.62 – 2.34), with significant differences by age group. The prevalence was highest in women aged 21–24 (6.12%; 95% CI 4.72 – 7.92) and declined steadily as women aged, to 0.48% (95% CI 0.25 – 0.94) in women aged 40–64 years (p_trend_<0.0001) (Table 2). Overall, the prevalence of co-infections was low (0.25% for *Mycoplasma genitalium* and *Chlamydia trachomatis* and 0.29% for *Mycoplasma genitalium* and *Trichomonas vaginalis*). Highest rates of *Mycoplasma genitalium* and *Chlamydia trachomatis* co-infection were in women aged 21–24 years (1.26%; 95% CI 0.70, 2.27) and declined significantly with increasing age (p_trend_=0.003) (Table 2). Similarly, highest rates of *Mycoplasma genitalium* and *Trichomonas vaginalis* co-infection were in women aged 21–24 years (0.78%; 95% CI 0.37–1.66%), with declining rates as age increased (p_trend_=0.012).

## Discussion

In this population-based study, we assessed the weighted population prevalence of *Mycoplasma genitalium* among women aged 21–64, attending routine cervical cancer screening in New Mexico, USA. We found an overall *Mycoplasma genitalium* prevalence of 1.95%, with highest rates among women aged 21–24 (6.12%). The majority of *Mycoplasma genitalium* infections across all ages occurred as single infections.

The observed prevalence of *Mycoplasma genitalium* was more than two-fold higher than the prevalence reported in a previous population-based US study in which asymptomatic individuals aged 18–27 years were tested (2.0% vs. 0.8%)(11), though the latter test used different tests and sample types which may have affected their test sensitivity relative to more recently validated tests. The prevalence in our study was much lower than prevalence rates based on individuals with symptoms or seeking care at STD clinics (2.0% vs. up to 26%) (12–14). The high *Mycoplasma genitalium* prevalence in the present study, particularly among the youngest women, is concerning as these estimates may reflect a significant number of asymptomatic women. If these infections remain undetected and untreated, these women may not only be at risk for future disease themselves but may also provide a reservoir for further transmission.

Among women aged 21–24 years, the prevalence of *Mycoplasma genitalium* was two-fold higher than the prevalence of *Chlamydia trachomatis* reported in the 2007–2012 National Health and Nutrition Examination Survey (NHANES) (6.1% vs. 2.9%) (15), but similar to the *Chlamydia trachomatis* prevalence in the present study (6.12 vs. 6.22). Whereas efforts are made to reduce the burden of *Chlamydia trachomatis* and associated disease by screening individuals (16), no efforts are currently in place to reduce the burden of *Mycoplasma genitalium*, despite that screening for STI may be efficacious at prevalence rates greater than 3.1% (17). Screening and treatment for *Chlamydia trachomatis* is associated with lower risk of pelvic inflammatory disease and infertility (2, 9, 18), but screening for *Mycoplasma genitalium* remains controversial, mainly because PID is more commonly attributed to *Chlamydia trachomatis* than *Mycoplasma genitalium* (8). However, these estimates were based on much lower prevalence rates. Well-designed prospective studies are needed to assess the potential impact of screening for *Mycoplasma genitalium* on reproductive harms (19).

The relatively low rate of co-infections between *Mycoplasma genitalium* and *Chlamydia trachomatis* may be partially explained by treatment effects on *Mycoplasma genitalium* in women with screen-detected *Chlamydia trachomatis* infections. This may also explain the reported increase in macrolide resistance for *Mycoplasma genitalium* over time, up to 42–69% in the US, with increased risk of resistance among individuals with co-infections, and with important differences by gender, race, and sexual orientation (14, 20–23). To reduce the burden of *Mycoplasma genitalium* and risk of treatment failure, increased awareness of this emerging pathogen and correct diagnostics are critical. Routine testing for *Mycoplasma genitalium* should be considered when women present with symptoms or clinical evidence of infection, particularly in case of treatment failure. Testing may also be considered prior to invasive procedures among women at high-risk of being infected, such as women undergoing surgical abortion, as this would allow for adequate diagnostics and treatment, which subsequently may reduce risk for postoperative complications, including PID (24). This may be critical, as the prophylaxis used in conjunction with surgical abortion (i.e. doxycycline) (25) does not confer antimicrobial eradication of *Mycoplasma genitalium*.

Our sampling frame reflects women attending cervical screening in New Mexico. While we believe that this provides a stronger population-based sample compared with clinic-based data, it cannot be taken as a true population-based sample. Prevalence rates may be underestimated as women not attending cervical cancer screening may have a higher prevalence of STI because of lower access to care and, possibly, shared risk factors. On the other hand, prevalence rates may be overestimated as women undergoing screening may have been screened due to symptoms. Finally, we cannot rule out that rates of *Chlamydia trachomatis* and *Mycoplasma genitalium* co-infections may have been underestimated, as we have no knowledge on previous testing and treatment of C*hlamydia trachomatis*.

## Figures and Tables

**Table 1.** Sampling fractions, intended and adjusted for missing values.

Intended Sampling Design						Actual Sampling Design^1^				
Cytology	Age	Intended		Sampling	Sampling	Age	Actual	Adjusted	Sampling	Sampling
	15 Strata	Sample	Population	Fraction	Weight	13 strata	Sample	Population^2^	Fraction	Weight
Normal	15–20	113	1529	7.39%	13.53					
	21–24	683	7589	9.00%	11.11	21–24	653	7589	8.60%	11.62
	25–30	1110	12472	8.90%	11.24	25–30	1,071	12472	8.59%	11.65
	31–34	334	8350	4.00%	25.00	31–34	318	8350	3.81%	26.26
	35–39	356	8476	4.20%	23.81	35–39	347	8476	4.09%	24.43
	40–44	333	8538	3.90%	25.64	40–44	323	8538	3.78%	26.43
	45–49	322	8474	3.80%	26.32	45–49	308	8474	3.63%	27.51
	50–54	356	9368	3.80%	26.31	50–54	343	9368	3.66%	27.31
	55–59	329	8436	3.90%	25.64	55–59	316	8436	3.75%	26.70
	60–64	267	6846	3.90%	25.64	60–64	251	6846	3.67%	27.27
	65+	201	5289	3.80%	26.31					
Total Normal		4404	85367				3930	78549		
ASCUS	all	2312	4624	50.00%	2	21 – 64	2,124	4396	48.32%	2.07
LSIL	all	732	1464	50.00%	2	21 – 64	635	1348	47.11%	2.12
ASC-H	all	223	223	100.00%	1	21 – 64	199	209	95.22%	1.05
HSIL	all	196	196	100.00%	1	21 – 64	170	184	92.39%	1.08
Total Abnormal		3463	6507				3128	6137		
TOTAL		7867	91874				7058	84686		

1. Actual sampling design adjusts for missing data

2. Population estimates as supplied by NMHPVPR adjusted to reflect routine screening age

The table summarizes details on the intended and actual sampling design fractions and resulting weights used in analysis to estimate population-level statistics. Actual sampling design weights in last column were used.

**Table 2.** Weighted prevalence estimates of *Mycoplasma genitalium* and *Chlamydia trachomatis* and *Trichomonas vaginalis* co-infections among women aged 21–64 years attending routine cervical cancer screening in New Mexico, USA

Age (years)	n	Mycoplasma genitalium prevalence % (95% CI)	Co-infection prevalence % (95% CI)	
			*Chlamydia trachomatis*	*Trichomonas vaginalis*
*All*	84,686	1.95 (1.62, 2.34)	0.25 (0.15, 0.43)	0.29 (0.18, 0.47)
*21–24*	8,765	6.12 (4.72, 7.92)	1.26 (0.70, 2.27)	0.78 (0.37, 1.66)
*25–30*	13,916	3.65 (2.78, 4.77)	0.12 (0.03, 0.49)	0.66 (0.35, 1.23)
*31–34*	9,069	2.72 (1.50, 4.87)	0.59 (0.15, 2.28)	0.05 (0.01, 0.18)
*35–39*	9,180	1.61 (0.78, 3.32)	0.05 (0.01, 0.18)	0.27 (0.04, 1.87)
*40–64*	43,756	0.48 (0.25, 0.94)	0.06 (0.01, 0.43)	0.13 (0.03, 0.48)

## Abbreviations

STI: sexually transmitted infection
STD: sexually transmitted disease
PID: pelvic inflammatory disease
HPV: human papillomavirus
LBC: liquid-based cytology
NMHPVPR: New Mexico HPV Pap Registry
